# Computed tomography-guided thoracoscopic debridement for multiple loculated organizing empyema: a case report

**DOI:** 10.1186/s40792-019-0731-5

**Published:** 2019-11-07

**Authors:** Masaya Aoki, Tadashi Umehara, Shoichiro Morizono, Yasuhiro Tokuda, Go Kamimura, Takuya Tokunaga, Souichi Suzuki, Aya Harada Takeda, Koki Maeda, Toshiyuki Nagata, Naoya Yokomakura, Kota Kariatsumari, Kazuhiro Ueda, Masami Sato

**Affiliations:** Department of General Thoracic Surgery, Kagoshima University Graduate School of Dental and Medical Science, 8-35-1 Sakuragaoka, Kagoshima, 890-8520 Japan

**Keywords:** Multiple loculated organizing empyema, Debridement, Video-assisted thoracoscopic surgery, C-arm cone-beam computed tomography

## Abstract

**Background:**

Video-assisted thoracoscopic surgery (VATS) for organizing empyema is challenging because fibrous septa and peel within the cavity are thickened and hardened. Some patients have multiple isolated empyema cavities that require debridement individually because firm intrathoracic adhesion was developed during this phase. If the debridement was incomplete as a result of worrying about an accidental injury of the surrounding organ, additional interventions may be required due to the persistent empyema cavity or insufficient expansion of the ipsilateral lung. We here describe a representative case with multiple loculated organizing empyema that could safely and reliably perform VATS debridement under C-arm cone-beam computed tomography (CBCT).

**Case presentation:**

A 67-year-old woman was admitted to our department for the treatment of right empyema. Chest computed tomography showed fluid collection in three independent spaces within the right thoracic cavity. It was assumed that a firm adhesion between the lung and chest wall was developed because about 7 weeks passed since the onset. Therefore, we decided to use CBCT to completely debride three empyema cavities separately by VATS. One cavity was only in a narrow range with the chest wall, and it was located on the back of cost rib cartilage. By clicking any intended anatomical structures on CBCT images, the position was readily depicted by lase projection on the body surface, which helped to place the best skin incision. Moreover, in other cavities, CBCT after initial debridement showed insufficiently dissected cavity. Additional debridement resulted in a successful shrinkage of the empyema cavity.

**Conclusion:**

We believe that VATS debridement under CBCT guidance is one of the useful treatment options for multiple loculated organizing empyema.

## Background

Over 50 years ago, the American Thoracic Society described three stages in the natural course of pleural empyema, namely the exudative, fibrinopurulent, and organizing phases [1]. In the fibrinopurulent phase, the empyema cavity is multi-chambered with fibrous septa. The septa at this phase are soft and it becomes a good indication for surgical debridement by video-assisted thoracoscopic surgery (VATS) [2, 3]. However, VATS for organizing phase empyema is challenging because fibrous septa and peel within the cavity are thickened and hardened. It sometimes is difficult to confirm the accurate extent that should be dissected, which can result in incomplete debridement or injury of vital organs [4, 5]. We here describe a representative case with multiple loculated organizing empyema who underwent VATS debridement under C-arm cone-beam computed tomography (CBCT; Artis zeego; Siemens Health GmbH, Erlangen, Germany) in a hybrid operating room.

## Case presentation

**Fig. 1:**
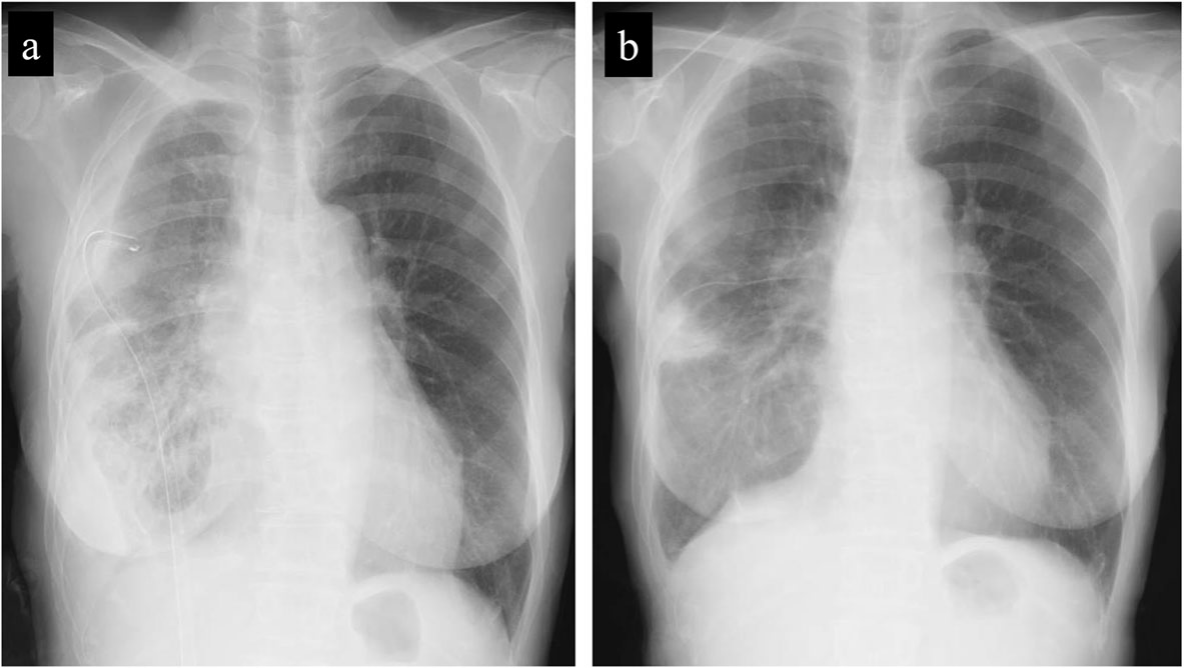
Chest X-rays at the time of admission and before discharge. Chest X-ray at the time of admission showed remaining pleural effusion and insufficient expansion of the lung, although a drainage tube had been inserted in the right thoracic cavity (**a**). Chest X-ray before discharge showed a slight shadow due to atelectasis, but well expansion of the other part of the lung (**b**)

**Fig. 2:**
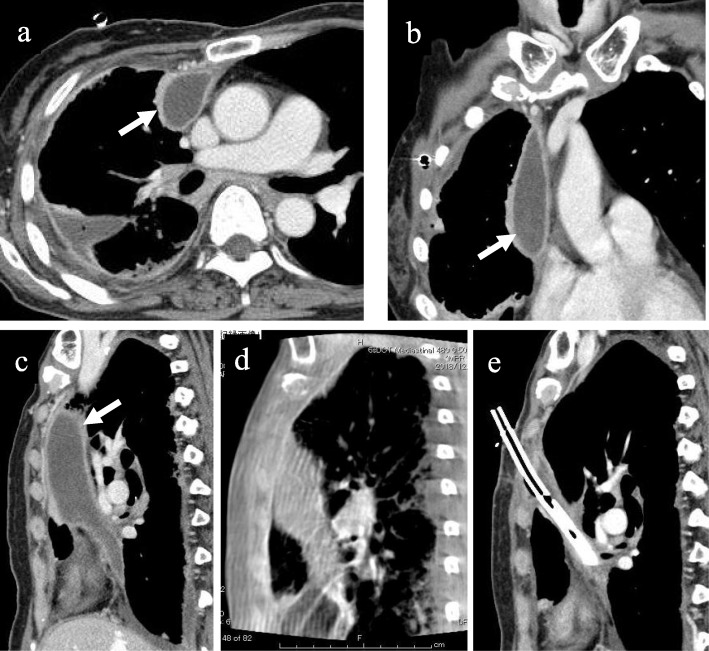
Cavity I: This cavity was from the ventral side of the superior vena cava in the anterior mediastinum to the inferior pulmonary vein along the pericardium. Axial view (**a**), coronal view (**b**), and sagittal view (**c**) of chest CT findings at admission. Intraoperative CBCT before (**d**) and after (**e**) the debridement and drainage of the organized empyema cavity. This empyema cavity was only in a narrow range with the chest wall, and it was located on the back of cost rib cartilage. Thus, we determined the location of skin incision using CBCT

**Fig. 3:**
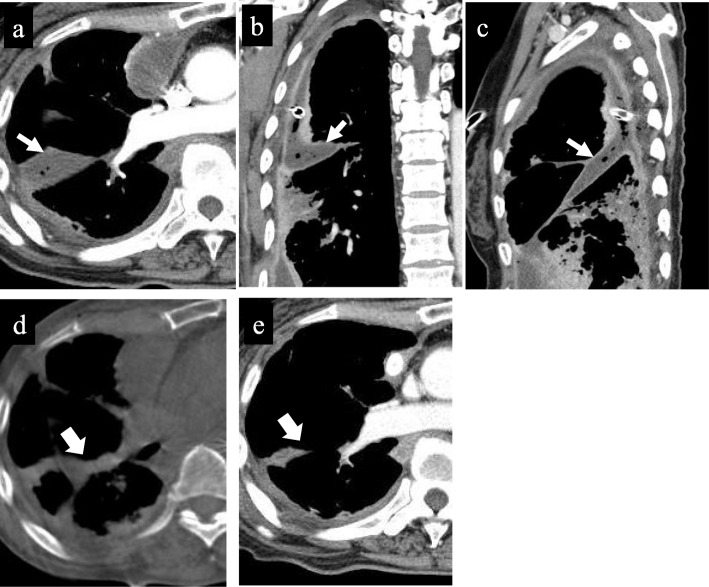
Cavity II: This cavity was present from outside of the right upper lobe to the interlobar space between upper and lower lobes, in which a chest drainage tube has been inserted. Axial view (**a**), coronal view (**b**), and sagittal view (**c**) of chest CT findings at admission. Preoperative CT shows empyema cavity with some air bubbles and thickened peel (**c**). Intraoperative CBCT after initial debridement shows insufficiently dissected cavity (**d**, arrow). Additional debridement resulted in successful shrinkage of the empyema cavity 2 weeks postoperatively (**e**, arrow)

**Fig. 4:**
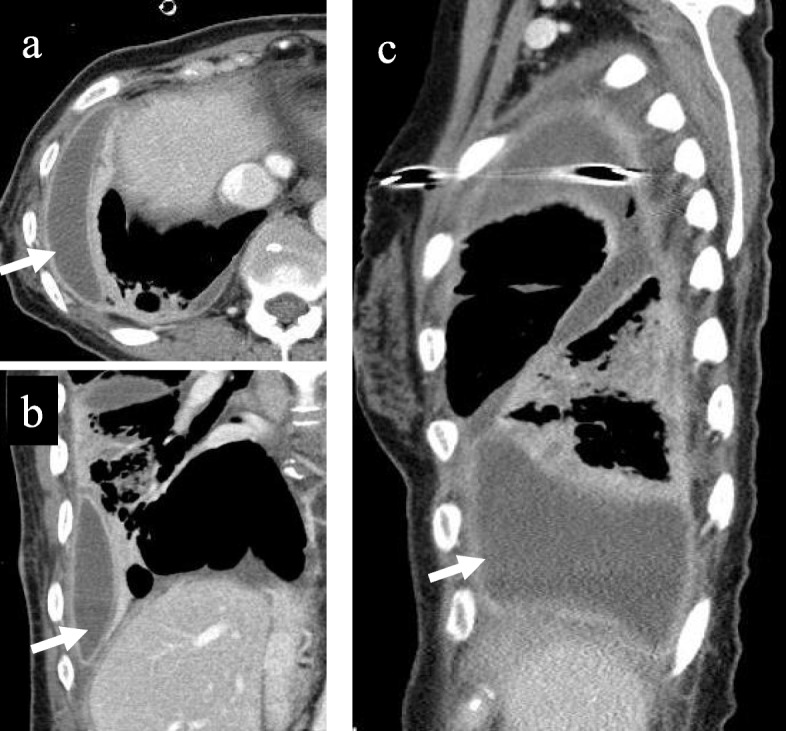
Cavity III: This cavity was located from the outside of the right lower lobe to the diaphragm. Axial view (**a**), coronal view (**b**), and sagittal view (**c**) of chest CT findings at admission. For this cavity, usual debridement under thoracoscope was possible without using CBCT

A 67-year-old woman was admitted to our department for the treatment of right empyema. She had been complaining of fever and fatigue about 7 weeks ago and had been treated with antibiotics by her primary care physician. However, as her symptoms did not improve, computed tomography (CT) was performed. A chest drainage tube was inserted because the chest CT showed pleural effusion with pleural thickening in the right thoracic cavity. She was diagnosed with empyema because bacteria were detected in cloudy fluid from the thoracic drainage tube. Blood and biochemical findings at admission showed an elevated inflammatory response (white blood cell 11520/μL and C-reactive protein 11.19 mg/dL). Chest X-ray showed remaining pleural effusion and insufficient expansion of the lung, although a drainage tube had been inserted in the right thoracic cavity (Fig. 1a). Chest CT showed fluid collection in three independent spaces within the right thoracic cavity. The first empyema cavity was from the ventral side of the superior vena cava in the anterior mediastinum to the inferior pulmonary vein along the pericardium (cavity I: Fig. 2a–c). The second empyema cavity was present from outside of the right upper lobe to the interlobar space between upper and lower lobes, in which a chest drainage tube has been inserted (cavity II: Fig. 3a–c). The third empyema cavity was located from the outside of the right lower lobe to the diaphragm (cavity III: Fig. 4a–c). It was assumed that a firm adhesion between the lung and chest wall was developed because a long time had passed since the onset. Therefore, we decided to use CBCT to completely debride three empyema cavities separately by VATS.

**Fig. 5:**
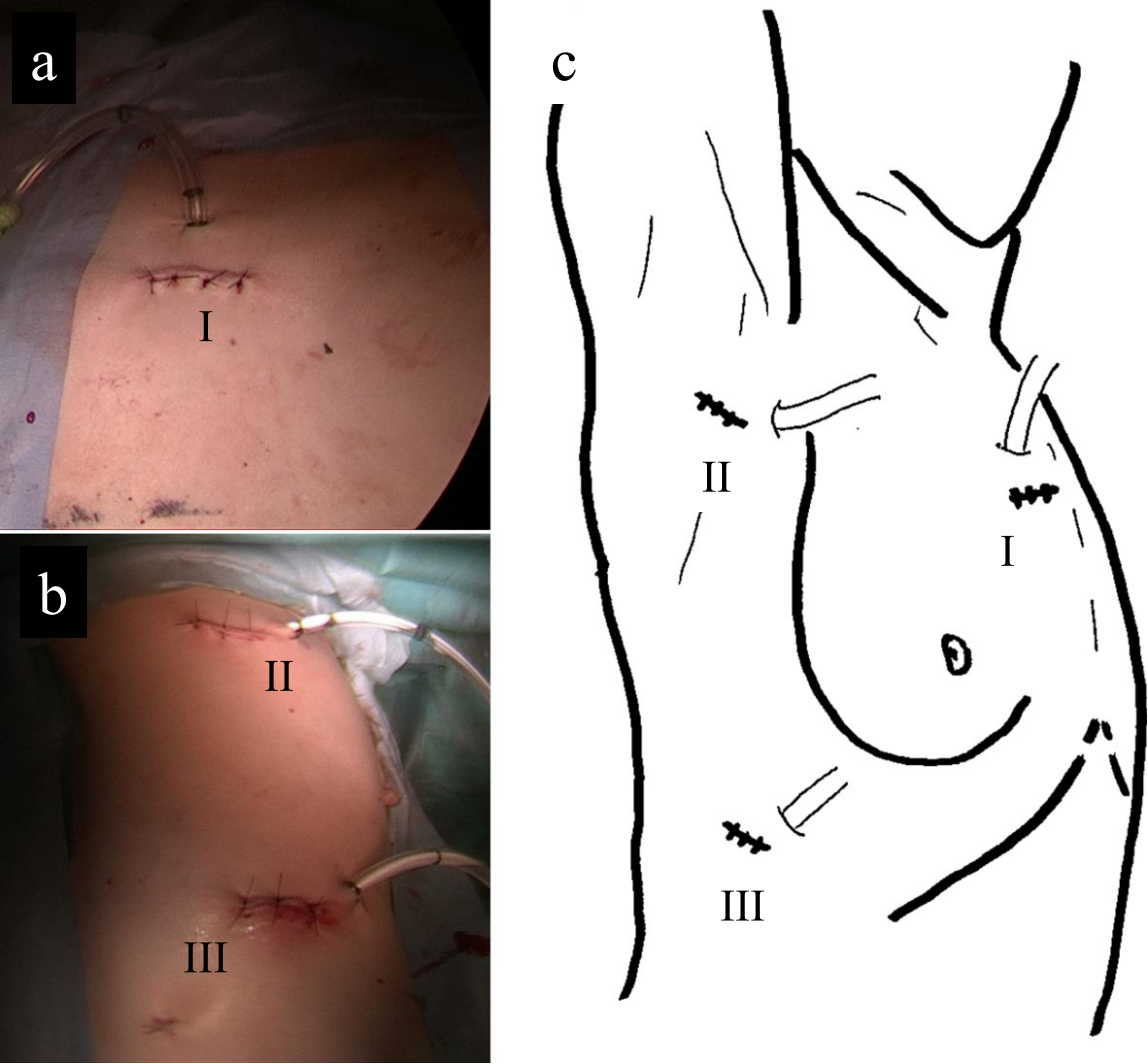
The pictures of three skin incisions and drainage tube (**a**, **b**) and their schema (**c**). Cavities I to III were debrided from the skin incision sites I to III, respectively

Under general anesthesia, the patient is placed in the lateral decubitus position. A careful check is made if the C-arm of the CBCT can be adequately rotated around the operation table. We can obtain scanning images of 18.5 cm in longer axis (craniocaudal direction) by a single rotation of the C-arm, and the single rotation needs 6 s (6 s acquisition protocol (6s DynaCT Body)). The field of view of the scanned image (24 × 24 cm, DynaCT mode) provided by CBCT is smaller than that of the multidetector computed tomography (MDCT). Thus, two rotations of the C-arm are needed to obtain images of the hemithorax. Scanning was performed under inflation of the lungs with 20 cm H_2_O airway pressure. The amount of irradiation dose in one rotation was 60 to 70 mGy. We took the images before and after the surgical procedure in order to determine the site of skin incision and to confirm the presence or absence of an overlooked empyema cavity. Cavity I was only in a narrow range with the chest wall, and it was located on the back of cost rib cartilage. By clicking any intended anatomical structures on CBCT images, the position was readily depicted by lase projection on the body surface, which helped to place the best skin incision (Fig. 2d, e). In cavity II, CBCT after initial debridement showed insufficiently dissected cavity (Fig. 3d). Additional debridement resulted in successful shrinkage of the empyema cavity (Fig. 3e). For cavity III, the usual debridement by VATS was possible without using CBCT. This patient had three isolated empyema cavities requiring chest drainage tube insertion into the individual cavities followed by VATS debridement using cupped forceps. The operation time was 315 min and the amount of bleeding was 225 g. A total of only three small incisions was made in this patient for adequate debridement of the three cavities (Fig. 5a–c). The patient recovered uneventfully without additional interventional therapy. Chest X-ray before discharge showed a slight shadow due to atelectasis, but well expansion of the other part of the lung (Fig. 1b).

CBCT in the hybrid operating room is currently used in various fields, such as cardiology, neurosurgery, and vascular surgery. With regard to respiratory surgery, although some reports describe the usefulness in identifying the intrapulmonary small lesions [6], it remains controversial if CBCT is useful in surgical treatment of empyema. Decortication via open thoracotomy requires wide adehesiolysis in order to peel the fibrous capsule of each cavity via single thoracotomy incision, which can often result in lung injury, thereby development of bronchopleural fistulae. Therefore, for some patients who have multiple isolated empyema cavities by developing firm intrathoracic adhesion, it is better performing debridement and drainage, individually. For these cases, VATS is advantageous because it does not necessarily require adhesiolysis and wide skin incision. However, concern remains whether the debridement without intrathoracic adhesiolysis is adequate or not because of the possibility of residual or overlooked contaminated cavities. Although transthoracic echo may also be useful to identify the location of the empyema cavity, this modality is not helpful to know whether the debridement is adequately accomplished or not. If the debridement was incomplete as a result of worrying about an accidental injury of the surrounding organ, additional interventions may be required due to the persistent empyema cavity or insufficient expansion of the ipsilateral lung [4, 5]. In the present case, accurate and effective VATS debridement and drainage with small skin incision was accomplished by using CBCT.

With respect to the radiation exposure, Chao et al. reported based on their experience with preoperative or intraoperative localization of small intrapulmonary nodules. They reported that the amount of radiation exposure by intraoperative localization with CBCT was comparable to that by preoperative localization with MDCT [6]. Because the amount of exposure by CBCT may depend on the times of C-arm rotation, we must attempt to restrict the times and extent of radiation exposure by CBCT.

## Conclusions

The best treatment strategy for multiple loculated organizing empyema remains controversial. We believe that thoracoscopic debridement under CBCT guidance is one of the useful treatment options for multiple loculated organizing empyema.

## Data Availability

All related data are included within the article.

## Abbreviations

CBCT: C-arm cone-beam computed tomography
CT: Computed tomography
MDCT: Multidetector computed tomography
VATS: Video-assisted thoracoscopic surgery
